# Effects of Hypertrophic and Dilated Cardiac Geometric Remodeling on Ejection Fraction

**DOI:** 10.3389/fphys.2022.898775

**Published:** 2022-05-31

**Authors:** Yu Zheng, Wei Xuan Chan, Christopher J. Charles, A. Mark Richards, Smita Sampath, Asad Abu Bakar Ali, Hwa Liang Leo, Choon Hwai Yap

**Affiliations:** ^1^ Department of Biomedical Engineering, National University of Singapore, Singapore, Singapore; ^2^ Department of Surgery, Yong Loo Lin School of Medicine, National University of Singapore, Singapore, Singapore; ^3^ Cardiovascular Research Institute, National University of Singapore, Singapore, Singapore; ^4^ Christchurch Heart Institute, Department of Medicine, University of Otago, Christchurch, New Zealand; ^5^ Translational Biomarkers, Merck Sharp & Dohme, Singapore, Singapore; ^6^ Department of Bioengineering, Imperial College London, London, United Kingdom

**Keywords:** ejection fraction, HFPEF, cardiac geometry remodelling, cardiac function, myocardial strain, mid-wall ejection fraction

## Abstract

**Background:** Both heart failure (HF) with preserved ejection fraction (HFpEF) and heart failure with reduced ejection fraction (HFrEF) can present a wide variety of cardiac morphologies consequent to cardiac remodeling. We sought to study if geometric changes to the heart during such remodeling will adversely affect the ejection fraction (EF) parameter’s ability to serve as an indicator of heart function, and to identify the mechanism for it.

**Methods and Results:** A numerical model that simulated the conversion of myocardial strain to stroke volume was developed from two porcine animal models of heart failure. Hypertrophic wall thickening was found to elevate EF, while left ventricle (LV) dilation was found to depress EF when myocardial strain was kept constant, causing EF to inaccurately represent the overall strain function. This was caused by EF being calculated using the endocardial boundary rather than the mid-wall layer. Radial displacement of the endocardial boundary resulted in endocardial strain deviating from the overall LV strain, and this deviation varied with LV geometric changes. This suggested that using the epi- or endo-boundaries to calculate functional parameters was not effective, and explained why EF could be adversely affected by geometric changes. Further, when EF was modified by calculating it at the mid-wall layer instead of at the endocardium, this shortcoming was resolved, and the mid-wall EF could differentiate between healthy and HFpEF subjects in our animal models, while the traditional EF could not.

**Conclusion:** We presented the mechanism to explain why EF can no longer effectively indicate cardiac function during cardiac geometric changes relevant to HF remodeling, losing the ability to distinguish between hypertrophic diseased hearts from healthy hearts. Measuring EF at the mid-wall location rather than endocardium can avoid the shortcoming and better represent the cardiac strain function.

## Introduction

Heart failure (HF) is the leading cause of morbidity and mortality worldwide, and the ejection fraction (EF) is widely adopted for evaluation of cardiac health and contractile function during HF. Clinically, it is an important parameter because of its association with and predictive power for cardiovascular outcomes (Solomon et al., 2005). However, it is known to have some shortcomings, such as variability between different imaging techniques, and only moderate correlation with overall cardiac function (Lupón and Bayés-Genís, 2018).

Currently, HF classification is based on EF. Classification into heart failure reduced ejection fraction (HFrEF), heart failure with mid-range EF (HFmrEF), or heart failure with preserved ejection fraction (HFpEF) is given for cases when EF ≤ 40%, 40% < EF<50%, and EF ≥ 50%, respectively. However, the inability of EF to indicate a decreased cardiac function in HFpEF and to classify it as diseased despite signs of HF points to its limitations and contributes to challenges in the diagnosis of HFpEF.

The EF’s inadequacy is likely related to geometric changes during HF cardiac remodeling. A wide range of cardiac geometric changes can occur during HF remodeling, and have been classified as concentric hypertrophy (CH), concentric remodeling (CR), and eccentric hypertrophy (EH), according to the LV mass (LVM) and relative wall thickness (RWT) (Nauta et al., 2020). Chamber dilation is also frequently observed in heart failure patients, especially those with eccentric hypertrophy (Zeng et al., 2017). Different types of Cardiac geometry remodeling are also reported in other heart diseases, like aortic stenosis (Di Nora et al., 2018). Further, investigators have found that specific geometric changes could increase or reduce the LVEF during the disease progression. MacIver et al. found that hypertrophic thickening of the myocardial wall, found in 48% of HFpEF (Katz et al., 2013), caused a compensatory increase in Left ventricle (LV) EF (MacIver et al., 2015) while Stokke et al. observed that cardiac dilation, which was found in 80% of HFrEF (Nauta et al., 2020), was responsible for a reduction in LVEF (Stokke et al., 2017). However, it remains unclear why LVEF is affected by geometric changes and what mechanism is responsible for this.

Currently, there is a contradictory observation between the LV global flow function indicated by the LVEF and LV myocardial contractility indicated by the global strains in HFpEF or hypertrophic LV patients. A few studies show that LV strain, especially the global longitudinal strain (GLS) could be a sensitive predictor for HFpEF than LVEF (Kraigher-Krainer et al., 2014; Hasselberg et al., 2015; Tadic et al., 2017; Potter and Marwick, 2018; Sanna et al., 2021), even in HFrEF patients (Mignot et al., 2010; Sengeløv et al., 2015; Park et al., 2018). Meanwhile, Kim et al.‘s study suggested that global circumferential strain (GCS) was a better predictor than GLS or global radial strain (GRS) in HFpEF (Kim et al., 2020). However, the mechanism behind the difference between LVEF and LV strain function during HF LV remodeling is clear.

In the current study, we seek to address these issues. We modelled cardiac hypertrophy and dilation using an idealized myocardial numerical model that was based on anatomic and strain measurements from porcine models of left ventricular hypertrophy and coronary artery disease (CAD), and we investigated the effects of the geometric changes during hypertrophy and dilation on LVEF and myocardial strain. We aimed to determine the mechanism by which geometric changes adversely affected the effectiveness of LVEF to indicate cardiac function and to recommend a modification to EF to correct this shortcoming.

## Methods

### Acquisition of Cardiac Magnetic Resonance Images

All animal studies were approved by the IACUC of National University of Singapore (protocol R15-0090). The LVH porcine model and MRI scans performed were reported in a previously publication, where further details can be found (Charles et al., 2020). Briefly, LVH model was induced in Yokrshire Landrace pigs (3–4 months old) by weekly incremental inflation of an aortic cuff to give a 20 mmHg increase in aortic pressure gradient every week, over a four-weeks period (up to 80 mmHg), starting from experimental day 7. CMRI images were obtained at baseline (pre-cuff placement or day 0), and at termination (42 days post-cuff placement) by the Skyra 3T MRI scanner (Siemens Medical Solutions, Erlangen, Germany). Images for 5 LVH subjects and 6 heart-healthy sham control subjects were obtained and analyzed. All animals in both groups were of similar age of between 3–4 months. Plasma B-type natriuretic peptide (BNP) levels was also assessed in 18 LVH subjects and 6 sham subjects, and was found to be significantly increased in LVH subjects, demonstrating HF (Charles et al., 2020). Only 5 LVH porcine subjects were chosen for study here as they were severe disease cases with the highest BNP values. 2D cine short-axis, 3-chamber and 4 chamber images were acquired.

CMRI was also obtained and analyzed in 10 female CAD porcine models (6–7 months old). In this model, HFrEF secondary to acute myocardial infraction was induced by permanent ligation of the left circumflex coronary artery (Timmers et al., 2007). MRI images were obtained at baseline (pre-CAD) and repeated at termination (28 days post-CAD) with the same scanner.

LV endocardial and epicardial boundaries were traced at both end-diastole (ED) and end-systole (ES) from both the long-axis and short-axis views to compute endocardial and epicardial strains, for both circumferential and longitudinal strains. Strains at the middle of the myocardial wall thickness, or the mid-wall location, was also obtained using the average length of the endocardial and epicardial boundaries. The inter-ventricular septal wall thickness (IVST), posterior wall thickness (PWT) and LV inner diameter at end-diastole (LVIDD) were measured from the short-axis view, and were used to calculate the relative wall thickness (RWT), as per (Lang et al., 2015):

$$RWT=\frac{IVST\;+\;PWT}{LVIDD}\;$$ (1)

The sphericity index (SI) was further calculated as the ratio of the basal-apical length to LVIDD at the mid-section, using the long-axis view. Supplementary Figure S1 demonstrated the examples of LV anatomic measurements.

LV mass (LVM) was calculated via the formula by (Devereux and Reichek, 1977) with a 20% correction as recommended by subsequent validation studies (Lang et al., 2015).

$$LVM=0.8\times 1.04\times \left[{\left(LVIDD+PWT+IVST\right)}^{3}-LVIDD^{3}\right]+0.6$$ (2)

The modified Simpson’s rule was adopted to estimate the LV volume (Folland et al., 1979):

$$Volume=\frac{A_{b}+A_{m}}{2}\times \frac{L}{3}+\frac{A_{m}+A_{p}}{2}\times \frac{L}{3}+\frac{1}{3}\times A_{p}\times \frac{L}{3}$$ (3)

Where A_b_, A_m_ and A_p_ were the cross-sectional luminal areas at the basal, one-third and two-thirds planes of the LV, calculated with diameter measurements and by assuming perfect circular transverse cross-sections. These approaches to obtain LVM and LV volume were used because MRI images were available only in 2D.

### Myocardial Numerical Model

**FIGURE 1:**
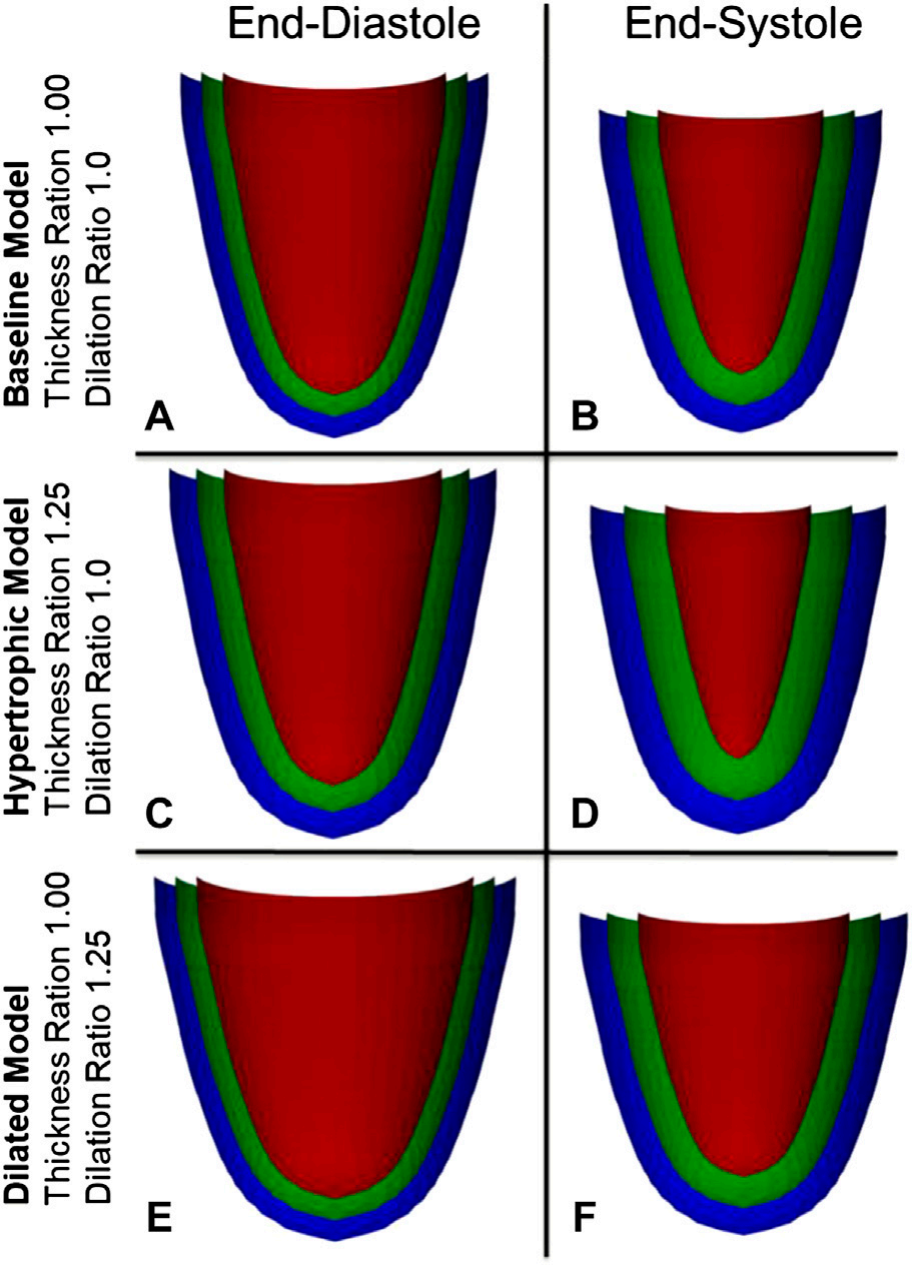
Visualization of the myocardial numerical model at three layers, the endocardium (red), the mid-wall layer (green), and the epicardium (blue), for the **(A,B)** normal heart, and during **(C,D)** myocardial wall thickening and **(E,F)** chamber dilation*.*

A simple 3D numerical model of the LV was developed to evaluate how specific myocardial strain magnitudes in specific cardiac geometries would translate to cardiac chamber volume changes, without any consideration of the fiber orientation, chamber pressure and myocyte force involved during contractions. The LV model was reconstructed as an idealized prolate shape, using radii and wall thickness measurements from the typical sham pig, and was visualized in Figure 1 with 3 myocardial wall layers, namely the endocardial, mid-wall and epicardial layers, where the half-thickness offset at ED between the endocardium and the mid-wall or between the epicardium and the mid-wall were the same. The mid-wall layer was modeled with 59 transverse circles that were equally spaced from the base to the apex, with varying number of nodes for each circle, ranging from 1 node at the apex to 38 nodes at the base. Equivalent meshes were generated for the endocardial and epicardial surfaces, but these were placed at specific radial offset from the mid-wall layer.

The mid-wall layer was programmed to exhibit spatially uniform ED-to-ES engineering strains, and could be programmed to be of varying strain magnitudes for both the longitudinal and circumferential strains, from −0.06 to −0.20 separately. This was achieved by adjusting the longitudinal distance between consecutive circles for longitudinal strain, and by adjusting the short-axis circumference for circumferential strain. The modelled contraction obeyed the physical property of tissue incompressibility, such that radial strains must be determined by the longitudinal and circumferential strains, and the endocardial and epicardial layers were adjusted in their radial offset from the mid-wall layer accordingly after contraction. Subsequently, changes to the epicardial and endocardial mesh were used to calculate the endocardial and epicardial strains. The ED and ES luminal volumes of the LV chamber were calculated via numerical integration for endocardial or traditional EF and SV calculations, and mid-wall ED and ES volumes were obtained to calculate the mid-wall EF.

To understand the effects of cardiac geometric changes to pumping function, increased wall thickness and increased LV diameter were modelled to the extent that was informed by our disease pig models. The reference geometry at end-diastole, which represents a healthy heart, was informed by the sham pig subjects, and had an RWT of 0.38, wall thickness of 7.14 mm, LVIDD of 37.62 mm, and sphericity index of 1.72 as shown in Figure 1A. Increased wall thickness was represented by the thickness ratio (range from 0.84 to 1.55), which was the multiple at which the wall thickness was increased from the reference geometry. When modelling wall thickening, the endocardium surface was fixed and kept constant. Increased LV diameter was represented by the dilation ratio (range from 0.80 to 1.40), which was the multiple at which LVIDD was increased from the reference geometry and wall thickness was constant.

For the current study, on top of quantifying EF, which was typically quantified at the endocardial boundary, we also quantified a modified EF that was quantified at the myocardial mid-wall layer. This was to evaluate if such an approach can improve this heart function parameter. To quantify this modified or mid-wall EF in the porcine models, we simply applied a correction factor ( α ) to the traditional endocardial EF, as follows.

$$\begin{array}{l} midwall\;EF=\;\alpha \times EF=\frac{Vol_{ED,endo}}{Vol_{ED,midwall}}\times \frac{SV}{Vol_{ED,endo}}=\frac{SV}{Vol_{ED,midwall}}\\ \alpha =\frac{Vol_{ED,endo}}{Vol_{ED,midwall}}=\frac{Vol_{ED,endo}}{Vol_{ED,endo}+\frac{LVM}{2\rho_{LV}}} \end{array}$$ (4)

Where *V*_*ED,location*_ was the end-diastole volume calculated at a location, either at the mid-wall or the endocardium, and $\rho_{LV}$ was the density of myocardium tissues of 1.05 g/ml. The correction factor, α , was essentially the ratio between the LV volume enclosed by the endocardial boundary and that enclosed by the mid-wall layer. This overall calculation essentially added half of the volume of the myocardium to the volume denominator in the traditional EF equation.

### Statistical Analysis

Normality of data was checked using the Anderson Darling test. For normally distributed data, 2-tailed t-tests were used for hypothesis testing, while for non-normally distributed data, the non-parametric Mann-Whitney test was employed.

## Results

### Cardiac Geometric Characteristics and Contractile Function in Porcine Models

**FIGURE 2:**
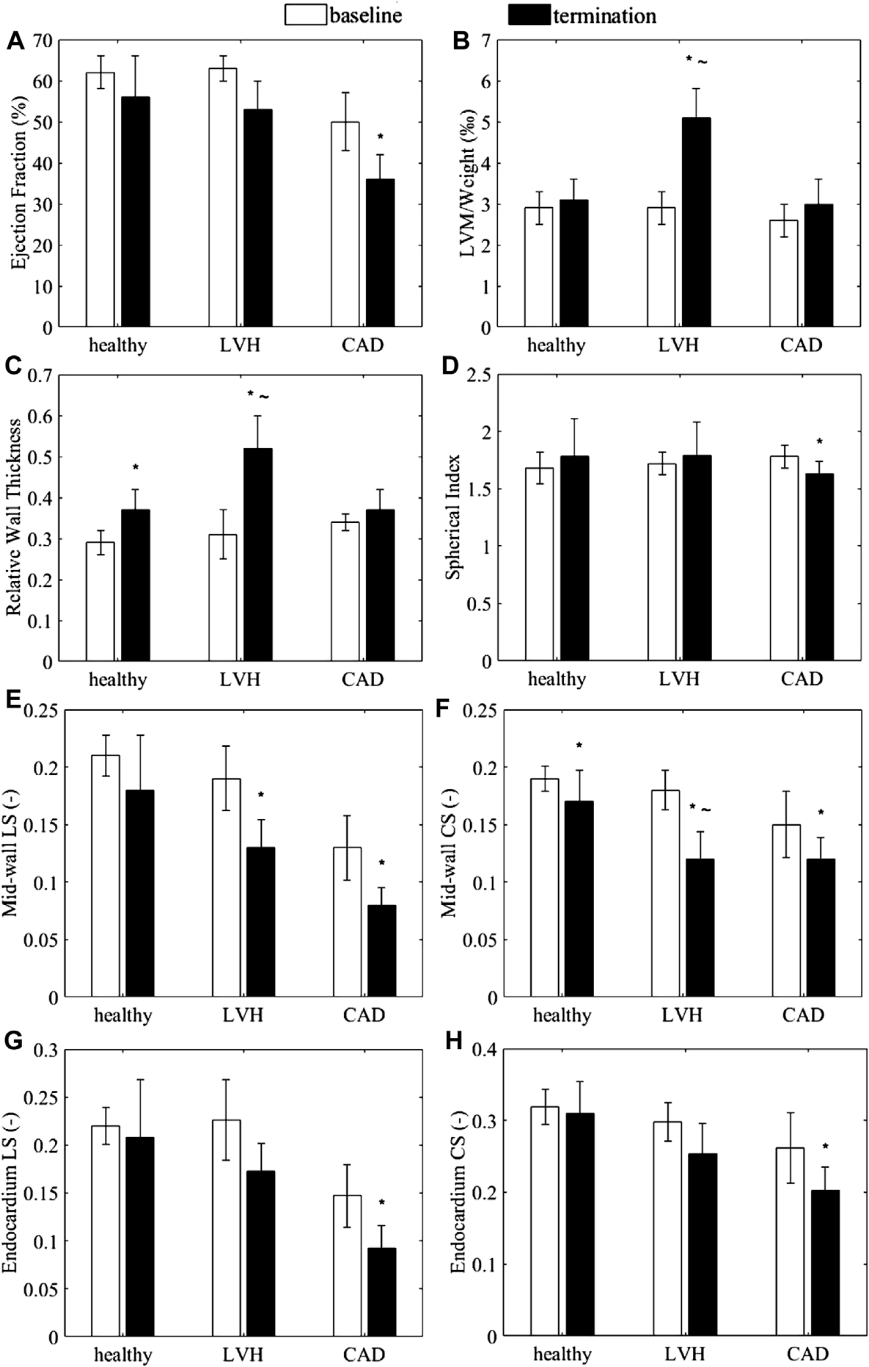
**(A–D)** Anatomic and functional characteristics of the porcine animal models, **(A)** Endocardial ejection fraction **(B)** LVM normalized by body weight, **(C)** relative wall thickness, and **(D)** spherical index. **(E–H)** Myocardial strains exhibited by the animal subjects. **(E,G)** Global longitudinal strain and **(F,H)** global circumferential strain at **(E,F)** the mid-wall and **(G,H)** the endocardium, measured from the healthy (sham), LVH and CAD porcine CMRI. *p < 0.05 with baseline. ∼ *p* < 0.05 with healthy for LVH only.

Geometric parameters, traditional EF and endocardial and mid-wall myocardial strains measured from the LVH and CAD models are presented in Figure 2. Other details, including wall thickness, SV and epicardial strain, are presented in the Supplementary Table S1.

EF for the LVH and control groups always exceeded 50% while EF in the CAD group were less than 40%, and BNP levels were significantly elevated in the LVH group (Charles et al., 2020). the LVH model could thus be considered to have HFpEF, while the CAD group could be considered as a model of HFrEF.

The LVH group demonstrated a significant increase in normalized LVM, RWT (Figures 2B,C), and wall thicknesses (Supplementary Table S1) compared to sham group at termination, but no change to sphericity index (Figure 2D), suggesting myocardial hypertrophy, which Katz et al. (2013) observed in 48% of HFpEF patients. On the other hand, the CAD group demonstrated no significant increases in LVM, RWT (Figures 2B,C), and wall thicknesses (Supplementary Table S1), suggesting no wall thickening, but demonstrated a significant decrease in the sphericity index, indicating a LV dilatation, which Nauta et al. (2020) also observed in 80% of human HFrEF patients. The average dimensions of the sham LVs were later used to construct the normal numerical heart model, while the measurements in the disease models were used to determine that extend of wall thickening (wall thickness ratio of 0.84–1.55) and LV dilatation (dilation ratio of 0.8–1.4) to investigate their effects on the cardiac function in our numerical modelling.

Figures 2E–H showed the mid-wall and endocardial longitudinal and circumferential strains in the animal models. For the LVH group, results showed that if strains were measured at the mid-wall location (Figures 2E,F), longitudinal strain was significantly less than its baseline, while circumferential strain were significant less than baseline and less than sham. However, these differences were reduced and were no longer significant when strains were measured from the endocardial location (Figures 2G,H). This could suggest that with wall thickening, the use of endocardial boundary for strain measurements could not reliably portray the decreased heart function, which our results below would reaffirm. On the other hand, for the CAD group without wall thickening, both circumferential and longitudinal strains were significantly lower at termination than at baseline on both the mid-wall and endocardial surfaces. This corroborated with the notion that wall thickening altered endocardial strains as a negative example.

### Effects of Wall Thickening and Dilatation Geometric Changes on EF and SV

Wall thickening and LV chamber dilatation were the two prominent cardiac remodeling features clinically and in our disease animal models. We thus investigated their effects on two cardiac function parameters, EF and SV, using our numerical model.

**FIGURE 3:**
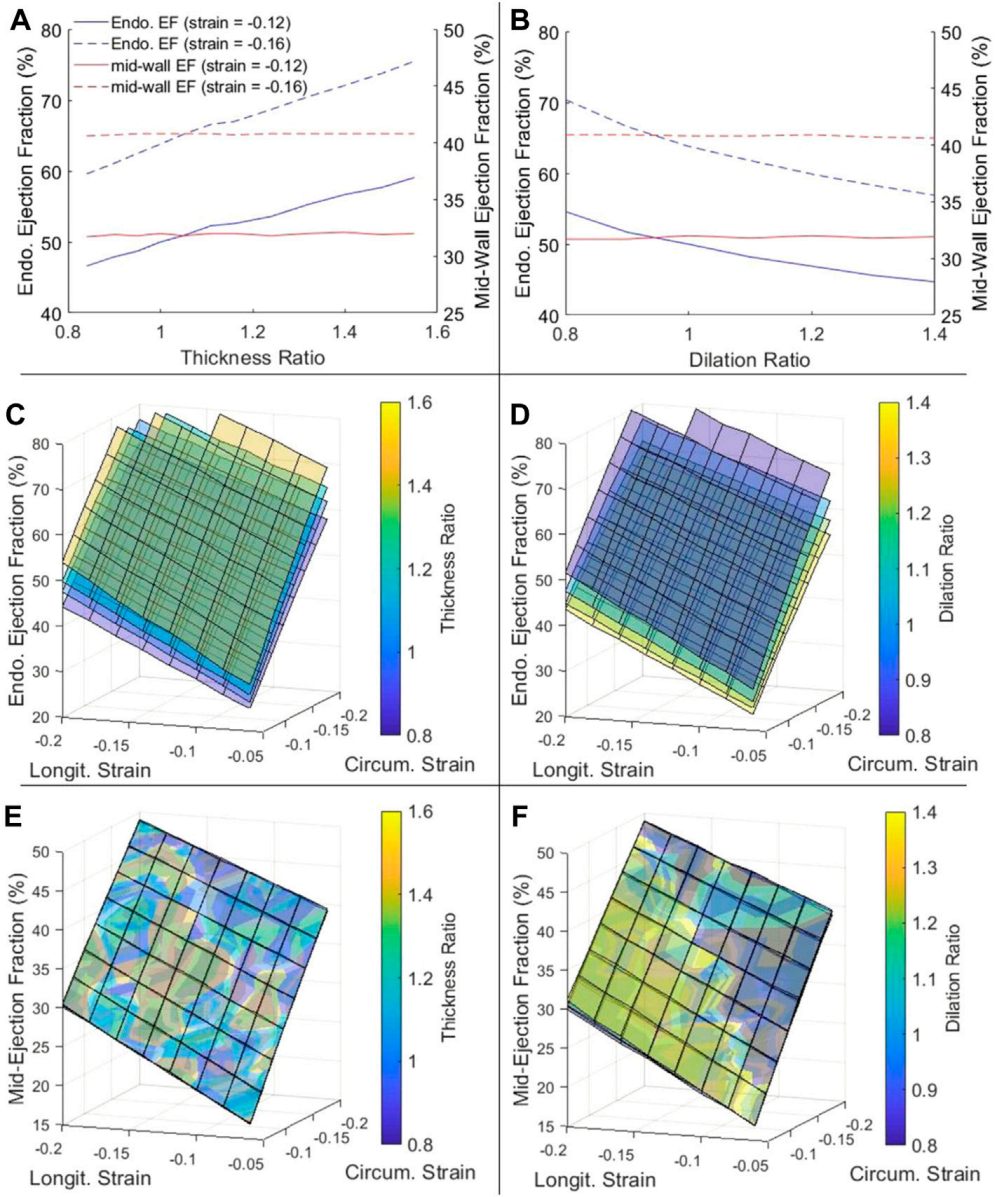
**(A,B)** The dependency of endocardial EF and mid-wall EF on **(A)** myocardial thickness ratio and **(B)** chamber dilation ratio, when strains at the mid-wall layer were fixed at either −0.12 or −0.16. **(C–F)** The complete dependency of **(C,D)** endocardial EF and **(E,F)** mid-wall EF on thickness ratio (four ratios from purple to yellow are 0.84, 1.00, 1.16 and 1.40), dilation ratio (four ratios from purple to yellow are 0.8, 1.0, 1.2 and 1.4), and longitudinal and circumferential strains at the mid-wall layer.

We tested effects of changing wall thickness and LV chamber dilatation with no change to contractile function, by keeping the mid-wall myocardial strain constant. Here, we ignored the fact that disease often reduced contractility, because we wished to isolate the effects of geometric changes in our model to understand its effects on EF and cardiac function, and we had thus kept all parameters other than geometric ones constant. Strains were kept constant at the mid-wall location as this is where global longitudinal and circumferential strains are typically measured. Results showed that, for the traditional endocardial EF that was measured at the endocardial boundary, increased wall thickness elevated EF (Figure 3A), while dilated LV chamber reduced EF (Figure 3B), which was not reflective of how strains did not change. However, if EF was measured at mid-wall, the mid-wall EF was constant despite changes to wall thickness and chamber dilation, thus providing a better representation of how strain was not changed. Alternations to the cardiac geometry that were common to HF cardiac remodeling thus adversely affected the endocardial EF to be unreflective of myocardial strain function, but the mid-wall EF was not affected by the same.

Figures 3C–F showed that this phenomenon held true even at different strain magnitudes. In Figure 3C, for example, when wall thickness ratio was changed, the relationship between strains and endocardial EF was changed and a new surface plot was needed to represent it, again suggesting that the endocardial EF was shifted when the geometry was changed. However, when mid-wall EF was calculated in Figure 3E, all the surface plots corresponding to different wall thicknesses collapsed into one unified behavior, suggesting that the mid-wall EF was independent of geometric changes. The same could be demonstrated for LV dilatation with Figures 3D,F.

Overall, Figures 3E,F showed that EF was sensitive to myocardial strains and should be able to represent strain function, but the mid-wall EF rather than endocardial EF was the better parameter to represent strain function as it was not shifted due to geometric changes.

**FIGURE 4:**
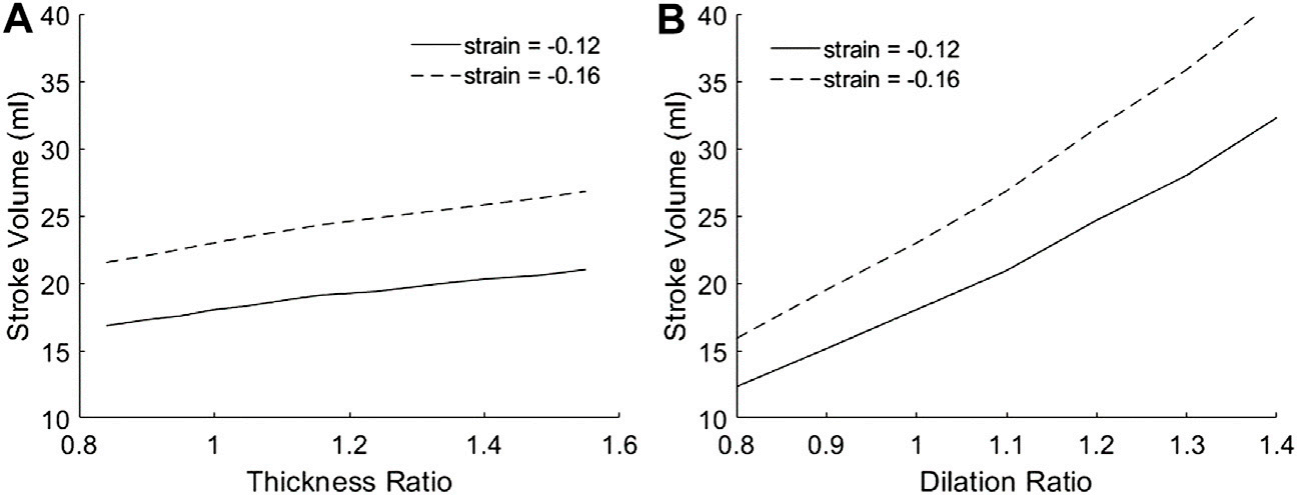
The dependency of SV on **(A)** myocardial thickness ratio and **(B)** chamber dilation ratio, when strains at the mid-wall layer were fixed at either −0.12 or −0.16. Results for other myocardial strains are given in Supplementary Figure S2.

Figure 4 showed the variation of SV with geometric changes, where the mid-wall strains was kept constant. Results shows that SV was slightly increased with wall thickening, but significantly increased with chamber dilation. This demonstrated that with geometric changes, the ability of the heart to convert strains to volumetric flow would be altered. For this reason, neither the endocardial EF nor the mid-wall EF performed well in representing the SV or the flow pumping function of the heart. However, the endocardial EF appear to be further off, as dilation reduced endocardial EF but increased SV. The complete dependency of SV on thickness ratio, dilation ratio, and longitudinal and circumferential strains was presented in Supplementary Figure S2.

We noted that Figure 4B demonstrated that dilation is geometrically advantageous to cardiac flow function, and could be a mechanism for compensating physiological stroke volume in the presence of hampered contractile function, such as during dilated cardiomyopathy. Past studies showed that such patients displayed similar stroke volume despite having contractile weakness (Eriksson et al., 2012; Nielles-Vallespin et al., 2017).

Results in Figure 4A were obtained by modelling wall thickening while keeping the endocardial boundary constant. i.e., the thickening was achieved by expanding the epicardial boundary only. Interestingly, when if wall thickening was modelled by keeping the mid-wall layer constant, i.e., expanding both the epicardial and endocardial boundary, SV would become independent of thickness ratio.

### Effects of Cardiac Geometry on Spatial Variability of Myocardial Strains

We further tested the effects of cardiac geometry on epi-to-endo spatial variability of strain, again by simulating various wall thickness and dilation ratios, but where the overall stain magnitudes, as indicated by the mid-wall strain, were kept constant.

**FIGURE 5:**
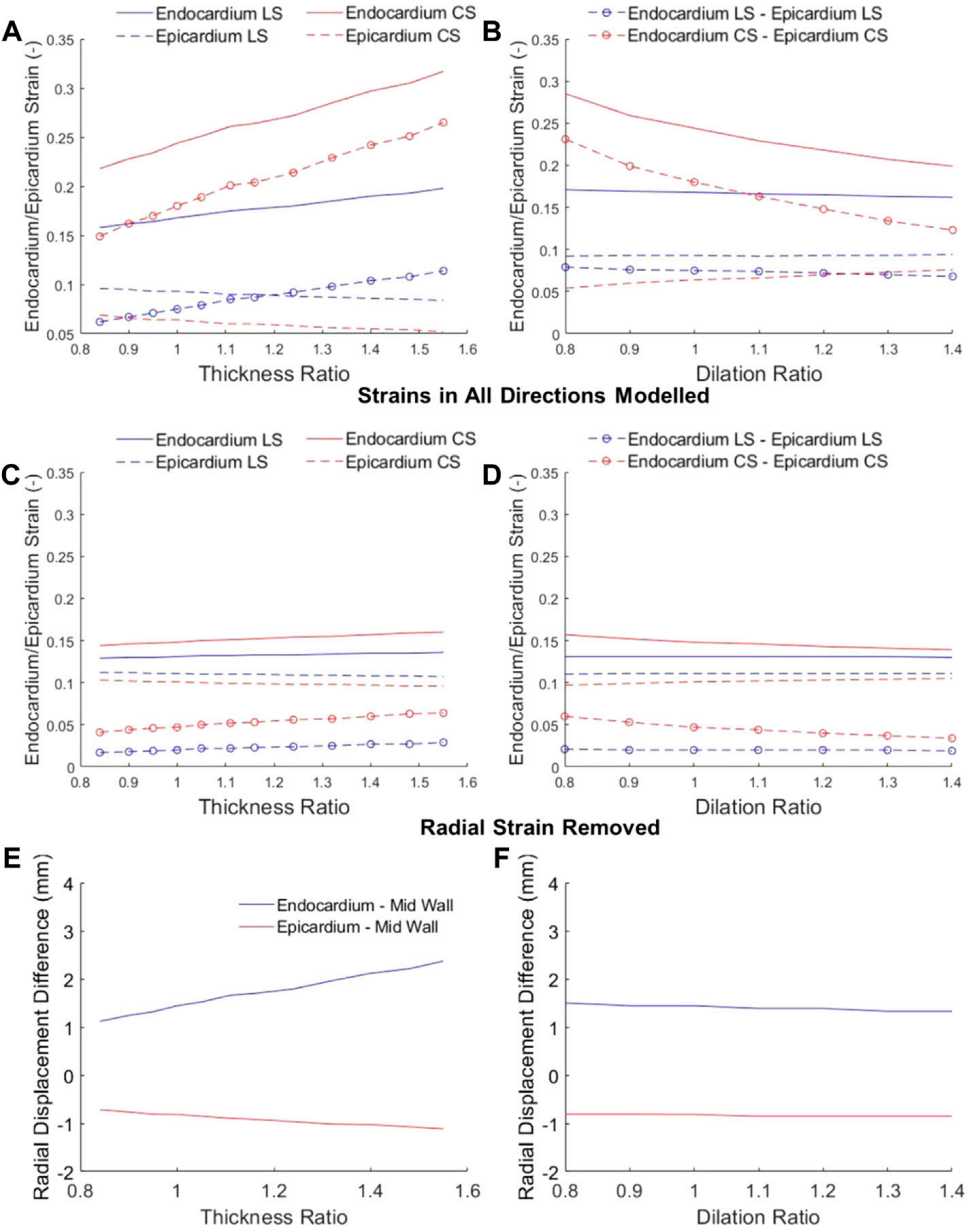
Longitudinal strain (LS) and circumferential strain (CS) at the endocardium and epicardium at various **(A,C)** wall thickness ratios and **(B,D)** dilation ratios, where the mid-wall ED-to-ES strains were kept constant at −0.12. In **(A)** and **(B)**, myocardium was appropriately modelled as incompressible, and LS and CS resulted in non-zero radial strains. In **(C)** and **(D)**, radial strains were assumed to be zero, to show that without radial displacements, there would be minimal epi-to-endo spatial strain variability. **(E,F)** the radial displacements of the epicardial and endocardial surfaces from the mid-wall when the heart has **(E)** wall thickening or **(F)** dilation.

Results showed that epicardial strains were lower than endocardial strains. Further, with increasing wall thickness, endocardial strain increased, but epicardial strain decreased, resulting in larger differences in strains at the two boundaries (Figure 5A). With chamber dilation, endocardial strain decreased but epicardial strain increased in the circumferential direction, thus decreasing the spatial variability, but strains at both locations remained relatively constant in the longitudinal direction (Figure 5B). These results are corroborated by clinical measurements of epi- and endo-strains, where the endo-to-epi strain ratio was found higher in hypertrophic hearts, but reduced in dilated hearts (Ozawa et al., 2015). These results showed that endocardial strains are easily altered by geometric changes and departed from the overall myocardial strains, and are thus not reliable indicators of strain function.

**FIGURE 6:**
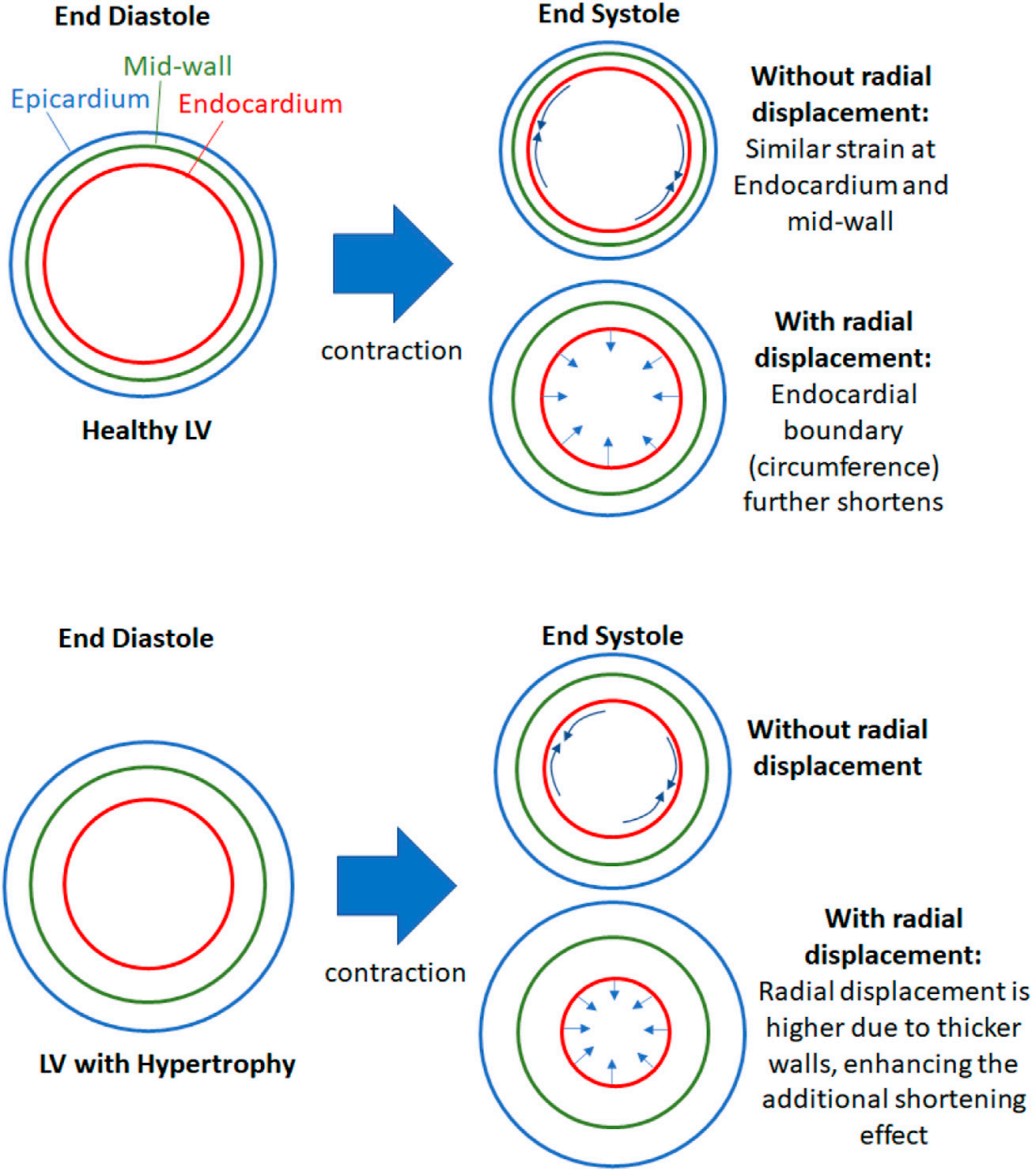
Schematic to explain the effects of radial displacement on endocardial strains. During contractions, the endocardial boundary not only shortens circumferentially (and longitudinally) but it is also pushed inwards by radial displacement, which shortens the endocardial boundary further. This additional shortening effect is enhanced when the heart is hypertrophic and the wall is thicker, since thicker wall will have higher radial displacement if the radial strain is not changed.

We propose that the reason for epi-to-endo difference in strains was due two factors, as depicted in Figure 6, the curvature of the myocardium, and the radial displacement during contractions. During systolic contractions, on top of longitudinal and circumferential shortening, the endocardial boundary was pushed inwards by radial displacement due to the wall curvature, and this added to the shortening of the endocardial boundary. Conversely, the epicardial boundary was pushed outwards during systolic contractions, and this negated some of the systolic shortening. The endocardial boundary would thus have higher strain than the epicardial boundary.

When the wall thickness was increased (Figure 6 lower panel), the same radial strain will cause larger radial displacements of the epicardial and endocardial boundary away from the mid-wall, thus enhancing the endo-to-epi spatial variability of strains, and worsening the deviation of endocardial strains from overall strains. Since endocardial strains are closely related to endocardial EF, endocardial EF is likely similarly affected and departed from overall strain function. This can explain the sensitivity of endocardial EF to wall thickening. When the LV was dilated, the radial displacements were not elevated, however, the curvature of the myocardial wall was reduced, and radial displacements did not add to endocardial shortening or negate epicardial shortening as much as before. This would thus decrease the epi-to-endo spatial strain variability, reduce endocardial strain’s overestimation of myocardial strain, and decrease endocardial EF.

To verify this mechanism, we first checked radial displacements in our numerical model in Figure 5E, and verified that when there were large radial displacements, epi-to-endo spatial variability of strains were higher (Figure 5A). Next, we removed radial strain from our numerical modelling, allowing only longitudinal and circumferential strains, by restricting the wall thickness to be constant at both ED and ES (Figures 5C,D), and found that the epi-to-endo spatial strain variability were mostly removed, and that strains became largely insensitive to geometric changes. These validated the role of radial displacement in influencing epi-to-endo spatial variability of strain and in enabling geometric changes to modulate endocardial strains.

**FIGURE 7:**
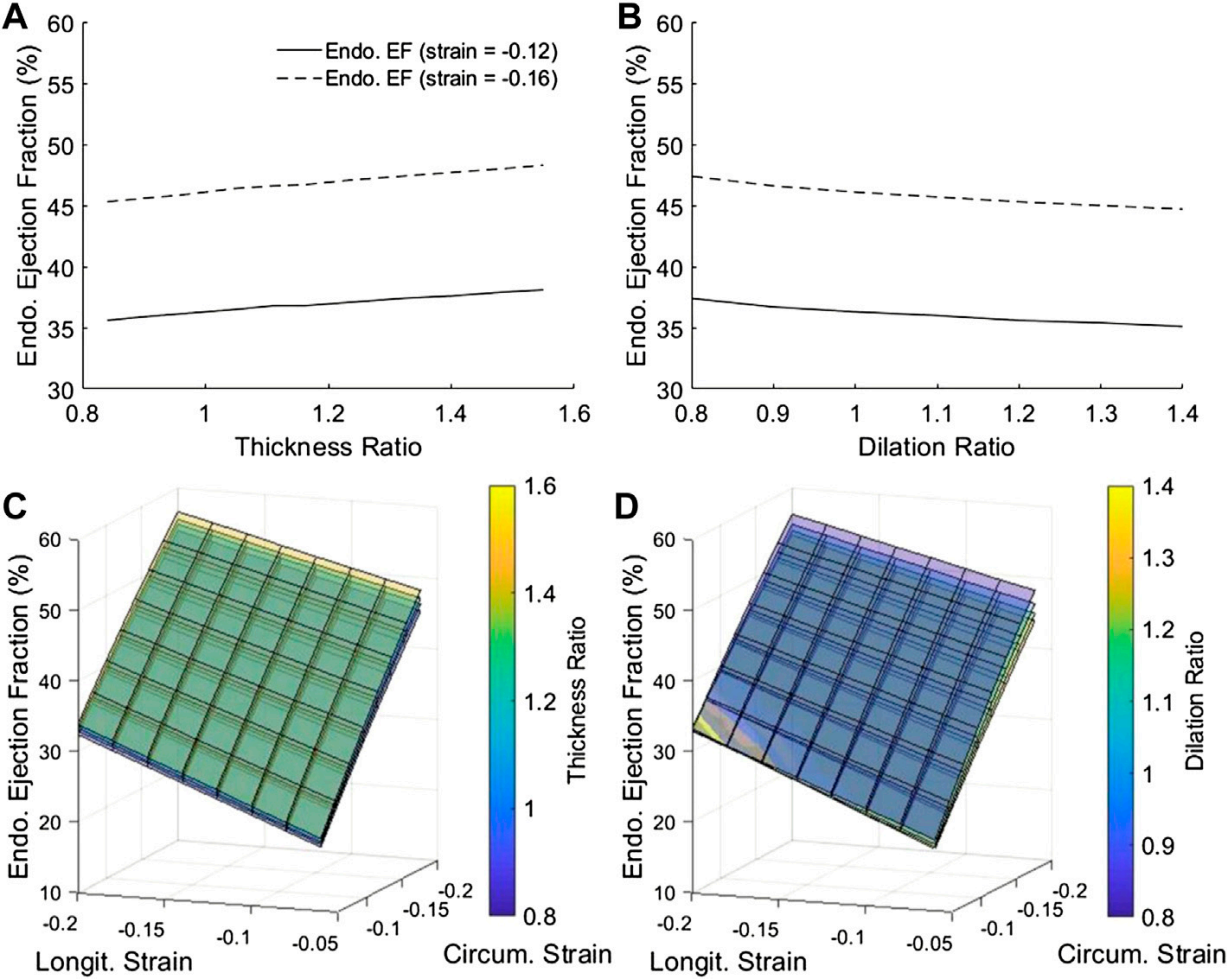
After removing radial strain from our numerical model, endocardial EF became largely insensitive to geometric changes, in contrast with results in Figure 3, demonstrating that radial displacement was the mechanism for endocardial EF shifting with geometry changes. **(A,B)** The dependency of endocardial EF on **(A)** myocardial thickness ratio and **(B)** chamber dilation ratio, when longitudinal and circumferential strains at the mid-wall layer were fixed at either −0.12 or −0.16, and when radial strain was 0. **(C–D)** The complete dependency of endocardial EF on thickness ratio, dilation ratio, and longitudinal and circumferential strains at the mid-wall layer.

Since endocardial EF is closely related to endocardial strain, we further tested whether this radial displacement mechanism was responsible for endocardial EF’s sensitivity to geometric changes. Figure 7 showed that by removing radial strain from the numerical model, the dependency of endocardial EF on geometric changes was mostly removed. This could be observed by comparing Figure 7 to Figures 3A,B. This thus validated the notion that radial displacement played an important role endocardial EF’s shifting with LV geometric changes.

### Endocardial EF versus Mid-Wall EF in Healthy, LVH and CAD Porcine Groups

Based on the above analyses, it followed logically that the mid-wall EF could resolve some limitations of the endocardial EF. First, at the mid-wall location, strains were close to the overall, average strain of the myocardium. Our animal data (Figure 2) showed that mid-wall strains could distinguish disease cases from healthy cases. Second, we showed that mid-wall EF was not sensitive to cardiac geometric changes, while endocardial EF was (Figure 3), which was likely due to mid-wall location not experiencing any radial displacement, which we showed was the mechanism for endocardial strain and endocardial EF’s shifting with geometric changes (Figures 5–7).

Mid-wall EF could be calculated by applying a correction factor, α , to the endocardial EF, as explained in Equation 4, and α could be calculated from LVM, which could be estimated by anatomic measurements via Equation 2. This process could thus be easily achieved with the standard clinical scans. We tested the mid-wall EF on CMIR images from our porcine animal models. Here, comparisons were made between the sham and LVH groups at termination, to determine the effects of LVH, since these were young and growing subjects (3–4 months old). For CAD, we compared CAD baseline to CAD termination, since CAD subjects were older (6–7 months old) than the other two groups and were no longer fast growing.

**FIGURE 8:**
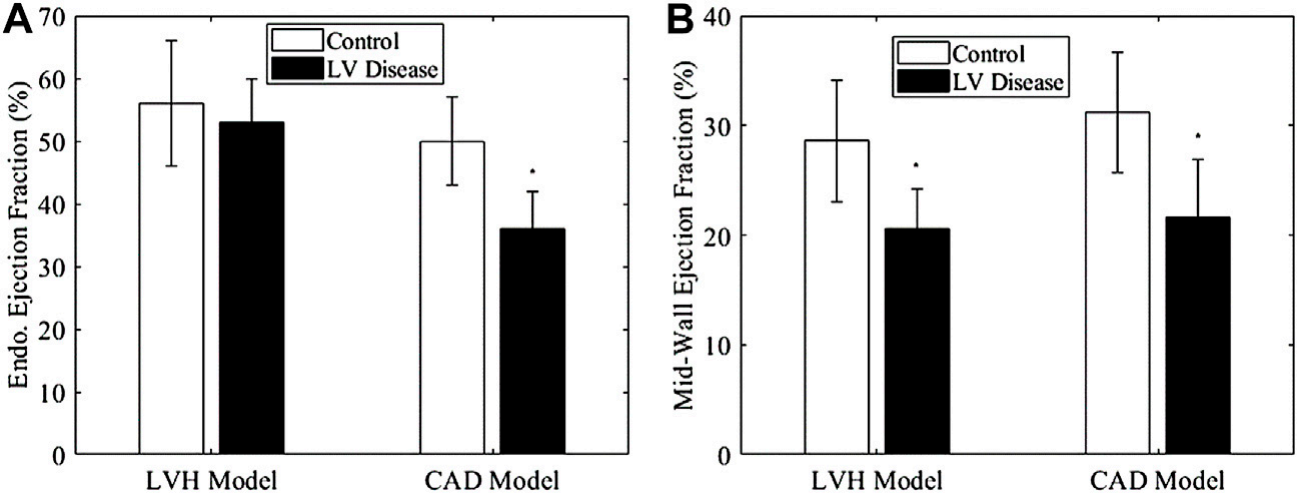
**(A)** Endocardial EF and **(B)** mid-wall EF of LVH model and CAD model at termination, compared to their appropriate controls. The control for LVH was sham at termination, while that for CAD group was its own data at baseline, Significance: *p < 0.05 comparing to the control group.

Results in Figure 8 showed that mid-wall EF successfully differentiated between the healthy (sham) and LVH groups, but the endocardial EF could not. This was because the LVH groups suffered from wall thickening (Figures 2B,C), which our modelling showed would artificially increase the magnitude of EF (Figures 3A,C) even when strain did not increase. Using the endocardial EF will thus result in the conclusion that EF is normal (>50%) when the heart is failing, in the same sense as the definition of HFpEF. Using the mid-wall EF, however, the differentiation of this LVH diseased group from the healthy group was possible. This might confer advantages during clinical evaluation.

For the CAD group, both endocardial EF and mid-wall EF were able to differentiate it from the healthy group. Thus, in this scenario, where there is no wall thickening, it did not matter which version of EF is used.

**FIGURE 9:**
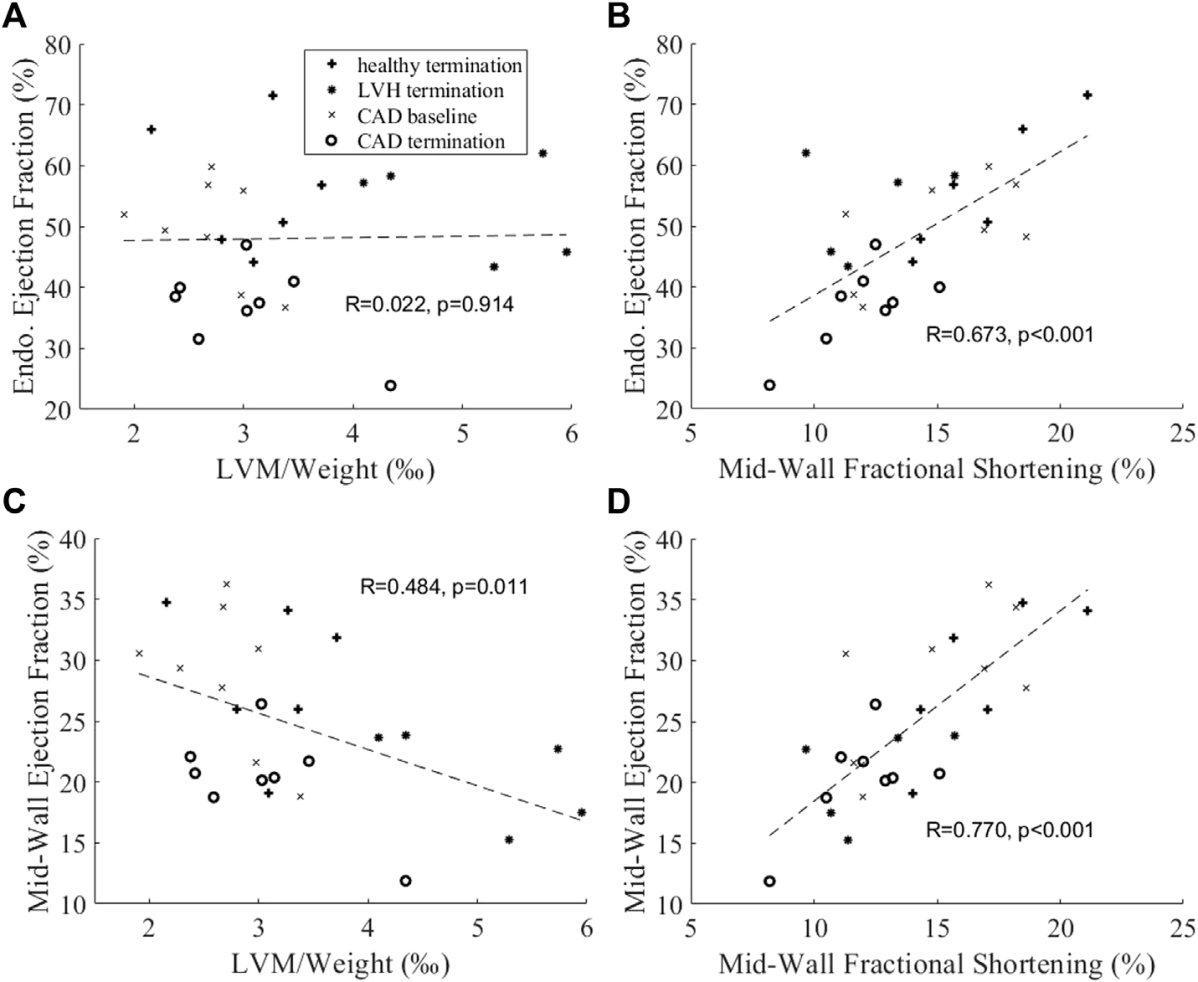
Correlation analysis between **(A,B)** endocardial EF or **(C,D)** mid-wall EF and **(A,C)** normalized LV mass and **(B,D)** mid-wall circumferential fractional shortening.

We further conducted correlation analysis with our animal data in Figure 9. Figures 9A,C showed that the endocardial EF did not correlate with normalized LVM, which was an indication of hypertrophic disease, but the mid-wall EF correlated negatively with it. This further indicated that the endocardial EF measure could not indicate hypertrophic decrease of cardiac function, while the mid-wall EF could. In Figures 9B,D, we further showed that the mid-wall EF correlated better with fractional shortening than the endocardial EF could, again suggesting that mid-wall EF could reflect strain function better.

Based on these promising results, we believe that the mid-wall EF could be a useful additional clinical parameter to evaluate heart failure cases, especially with hypertrophic cases. However, future studies are necessary to determine the utility and prognosis value of this EF measurement approach.

## Discussions

In our study, we demonstrated the mechanism for which the traditional endocardial EF parameter would be shifted by cardiac geometric changes (wall thickening and dilation), and which caused endocardial EF to be an ineffective measure of cardiac function during HF remodeling. We briefly summarize it here. The endocardial EF was closely related to myocardial strains at the endocardial surface, because EF described changes to the volume bound by the endocardial surface, while endocardial strain described changes to the surface area bound by the same surface. However, endocardial strains have a systemic deviation from the overall, average myocardial strain, because the endocardial surface would experience an inward radial displacement (caused by radial strain) that would elevate strains to be higher than the overall strains (Figure 6), and this elevation was dependent on wall thickness and curvature of the walls (Figure 5), because thicker walls will have higher radial displacement for the same radial strain magnitude. This thus caused endocardial strain to be sensitivity to geometric changes, and caused endocardial EF to be similarly sensitive as well (Figures 3, 7).

We validated this mechanism in a few ways. We showed that larger radial displacements during LVH corresponded to a larger deviation of endocardial strain from overall strain (Figures 5A,E), while reducing the wall curvature during dilation reduced this deviation (Figures 5B,F). We showed that by removing radial displacement, this deviation was much smaller (Figures 5C,D), and endocardial strain (Figures 5C,D) and endocardial EF (Figure 7) became much less sensitive to geometric changes than it originally was.

We believe that this mechanism can explain the clinical observations made by (MacIver et al., 2015) and Stokke et al. (2017), on how LVH artificially enhanced LVEF while dilation artificially decreased LVEF, and observations by (Ozawa et al., 2015) where hypertrophic cardiomyopathy hearts were observed to maintain their endocardial GCS despite epicardial and overall strains showing significant decrease from control hearts. Importantly, our results suggested that these shortcomings of endocardial EF were due to an inappropriate reliance on the endocardial boundary for quantifications, as the behavior of the endocardial boundary could not properly represent overall myocardial behavior, and departed from overall myocardial behavior in a geometric dependent way.

For this reason, we advocate the use of measurements at the mid-wall location for quantification of cardiac function. We showed in our study that mid-wall strains (Figure 2) and mid-wall EF (Figure 8) retained the ability to distinguish LVH porcine subjects from healthy ones when endocardial strains and endocardial EF could not, that mid-wall EF was not sensitive to cardiac geometric changes (Figure 3) but endocardial EF was, and that mid-wall EF correlated to severity of hypertrophic disease in our animal model when endocardial EF did not (Figure 9). We proposed a simple way to convert endocardial EF to mid-wall EF with existing echo or MRI measurements Eq. 4. In fact, our result concerning mid-wall EF was corroborated by past studies. Jung et al. and Yoshikawa et al. both measured the ejection fraction at the mid-wall and endocardial locations using echocardiography, and found that mid-wall EF distinguished LV hypertrophy group from the control group but endocardial EF could not (Jung et al., 2006; Yoshikawa et al., 2012).

In fact, our proposed use of mid-wall EF is equivalent to modifying EF to be more closely related to mid-wall myocardial strains, given the close relationship between EF and strains explained above. We propose that this is a good way to gauge cardiac health during HF remodeling, and there was support of this notion in the literature. Stokke et al. (2017) had concluded that cardiac strain reflects systolic function better than endocardial EF in patients with preserved EF, due to these geometric confounders (Faggiano et al., 2021). concluded that LV strain in patients with dilated LV chamber was a sensitive predictor without the obvious compensatory caused by the wall thickening. We propose that using mid-wall EF can address the current gap found between global contractility and EF in HF patients, especially in HFpEF (Mignot et al., 2010; Sengeløv et al., 2015; Park et al., 2018; Potter and Marwick, 2018). We believe that further clinical studies to validate this notion, and to investigate if mid-wall EF has more accurate prognosis value is warranted.

In our animal studies, it should be noted that the sham and LVH groups were young pigs (3–4 months old) that were less than 40 kg in body weight, and were thus fast-growing individuals. For this reason, LV mass, body weight, and myocardial strains would experience a natural reduction with growth before becoming stable after 40 kg of body weight, as demonstrated by (Rosner et al., 2008). This explained why even the sham group had decreased strains from baseline to termination, which was significant in terms of mid-wall circumferential strain (Figures 2E,F). Therefore, comparisons were performed between sham termination and LVH termination for the LVH model. The CAD group, however, were 6–7 months old, and were about 50 kg in body weight. They thus did not have the growth confounding factor, and since they were older than the sham group, comparisons were made between CAD baseline and termination.

### Limitations

One limitation of the study was the limited sample sizes of sham and LVH porcine CMRI datasets. A second limitation was that the LVH porcine model was performed in adolescent subjects, resulting in significant myocardial thickness and overall strain changes from baseline to termination even in the healthy group. The third limitation was that the CAD models were older than LVH models, and CAD did not have a sham control group. However, since the growth of CAD subjects during experiment was not significant, CAD baseline group could be used as the heart healthy control. The fourth limitation was that the numerical model was a simplified one based on idealized LV geometry, and this precluded modelling of subject specific LV geometries and fiber orientations and pressures, which would require a finite element model. Minor subject-specific shape variability of the hearts might thus have caused minor errors.

## Conclusion

Our animal model study and numerical modelling showed that the traditional EF, measured at the endocardial boundary, was an imperfect indicator of cardiac function because it was modulated by cardiac geometry changes during diseased cardiac remodeling, because of the reliance on the endocardial boundary for its calculation. We further showed that the mechanism for this modulation was that the endocardial boundary experienced radial displacements that caused its strain to deviate from strains elsewhere in the heart, and caused it to be unrepresentative of the strain function of the heart. This deviation was dependent on cardiac geometry, and explained why endocardial EF would be adversely affected by geometric changes. We further showed that by measuring EF at the mid-wall location, the mid-wall EF could represent the overall strain function of the heart better, and would no longer be affected by cardiac geometry changes. Further, the mid-wall EF could effectively distinguish LVH hearts from healthy hearts.

## Data Availability

The original contributions presented in the study are included in the article/Supplementary Material, further inquiries can be directed to the corresponding author.
