# Supplementary material for: Effects of Hypertrophic and Dilated Cardiac Geometric Remodeling on Ejection Fraction
Source: Front Physiol. 2022 May 31;13:898775. doi: 10.3389/fphys.2022.898775 (PMC9193973; doi:10.3389/fphys.2022.898775)
Supplement: Supplementary file 1 [file DataSheet1.docx]

**Supplementary Text**

**Measurements of Cardiac Dimensions in the Animal Models**

An example of the measurements of the myocardial short axis circumference, long-axis length, PWT, IVST, and LVIDD are shown in figure S1 below.


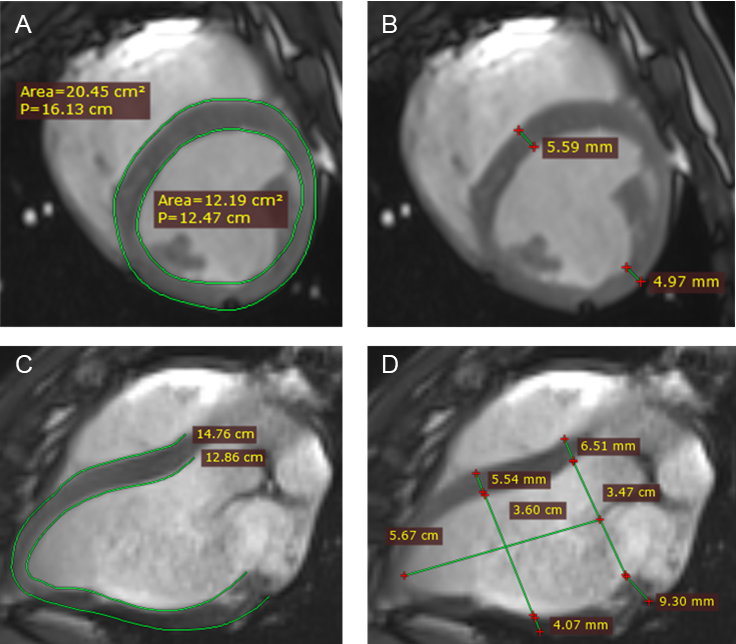


***Figure S1:*** *Manual measurement of MR geometric features in short-axis (A and B) and long-axis (C and D) MRI images.*

| **Group** | **Basal-Apex length/cm** | **LVIDD/cm** | **IVST/cm** | **PWT/cm** | **SV/ml** | **Epicardial LS** | **Epicardial CS** |
| --- | --- | --- | --- | --- | --- | --- | --- |
| **healthy (baseline)** | 5.85±0.32 | 3.89±0.10 | 0.59±0.03 | 0.55±0.07 | 24.5±1.8 | -0.19±0.02 | -0.10±0.01 |
| **healthy (termination)** | 5.68±0.58 | 3.91±0.19 | 0.71±0.12 | 0.74±0.07* | 22.3±6.5 | -0.16±0.04* | -0.07±0.01* |
| **LVH (baseline)** | 5.94±0.21 | 3.88±0.09 | 0.64±0.16 | 0.57±0.06 | 25.3±2.0 | -0.16±0.02 | -0.09±0.02 |
| **LVH (termination)** | 6.33±0.62 | 4.26±0.24*~ | 1.10±0.16*~ | 1.10±0.14*~ | 26.0±6.4 | -0.10±0.04*~ | -0.04±0.02*~ |
| **CAD (baseline)** | 7.65±0.47 | 4.82±0.28 | 0.81±0.07 | 0.81±0.08 | 44.5±12.6 | -0.12±0.03 | -0.07±0.02 |
| **CAD (termination)** | 7.34±0.44 | 4.89±0.36 | 0.91±0.08 | 0.92±0.16 | 35.2±9.0 | -0.07±0.02* | -0.06±0.02 |

***Table S1.*** *Cardiac anatomic and functional parameters measured from LVH, CAD, and healthy control porcine groups.*

LVIDD – LV inner diameter at end-diastole, IVST – interventricular septal wall thickness, PWT – posterior wall thickness, SV – stroke volume, epicardial LS – epicardial longitudinal strain, epicardial CS – epicardial circumferential strain. * p<0.05 in comparison with baseline. ~ p<0.05 with healthy group.


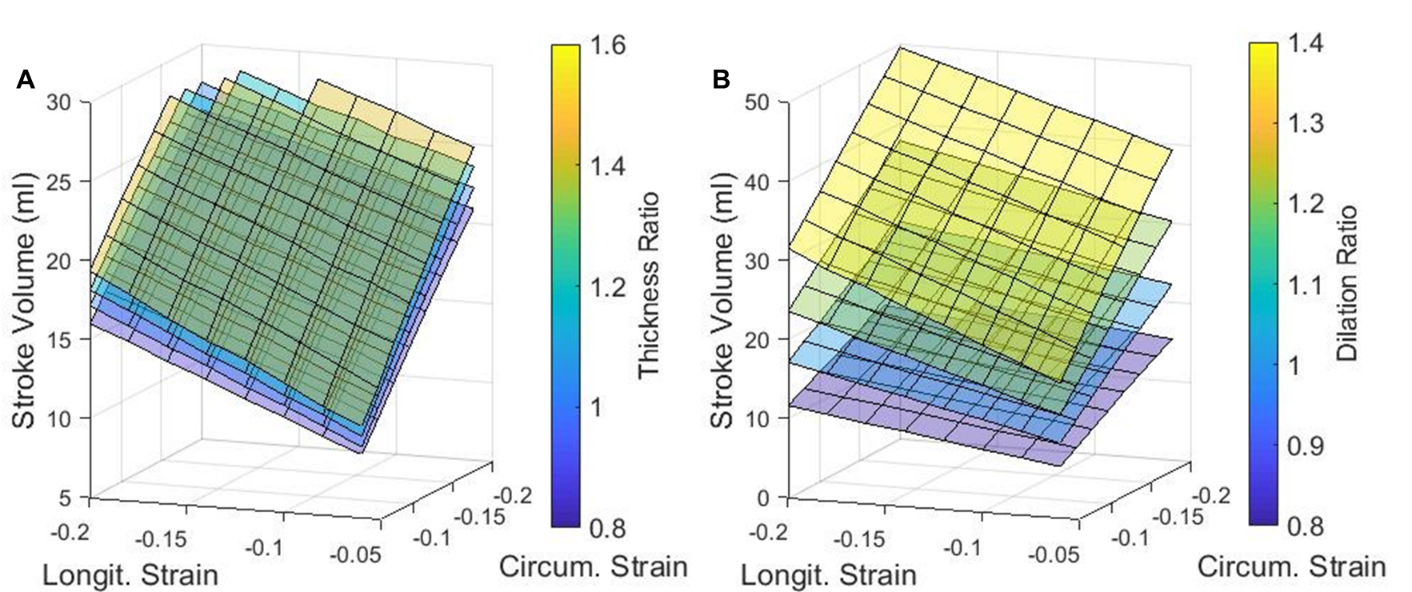


***Figure S2:*** *The dependency of SV on longitudinal and circumferential strains*

*for various (A) thickness ratio, (B) dilation ratio*
